# Arteriovenous fistula after radial catheterization with cardiopulmonary repercussions

**DOI:** 10.1590/1677-5449.008618

**Published:** 2019-01-30

**Authors:** Alexandre Faraco de Oliveira, Alexandre David Ribeiro, Marcio Costa Silveira Ávila

**Affiliations:** 1 Universidade do Planalto Catarinense – UNIPLAC, Lages, SC, Brasil.; 2 Clínica Ana Carolina, Lages, SC, Brasil.

**Keywords:** arteriovenous fistula, cardiac catheterization, percutaneous coronary intervention, dyspnea

## Abstract

This article describes the case of an 86-year-old coronary disease patient who underwent cardiac catheterization via a left radial access. Around 16 months after the procedure, he presented with dyspnea, unrelated to effort, but associated with nocturnal hypoxia. There was a palpable thrill in the left wrist and he was diagnosed with a radiocephalic arteriovenous fistula in the left wrist. A duplex scan revealed an abnormal wave pattern and increased diastolic velocity compatible with arteriovenous fistula. The fistula was repaired surgically and the patient exhibited improvement in clinical and laboratory parameters after the procedure. Radial access is increasingly being used for cardiac catheterization, primarily because it is associated with fewer and less harmful complications than femoral access. However, complications such as arteriovenous fistula occur and can be particularly harmful in octogenarian patients.

## INTRODUCTION

 Radial access is increasingly used for cardiac catheterization, whether diagnostic or therapeutic, and is now considered the preferred approach for the majority of applications. 1 Preference for the radial approach is primarily due to the lower incidence of complications after the procedure and reduced bleeding during the procedure. 2

 While complications related to radial access are undoubtedly less frequent, they do occur and can be especially harmful to older patients. They can be detrimental to control of the cardiac and pulmonary pathologies that often preexist access in this group of patients. 3 In the present article, we describe the case of a coronary disease patient who underwent cardiac catheterization and coronary angioplasty and developed an arteriovenous fistula (AVF) in the wrist. Around 1 year later he suffered dyspnea unrelated to effort, but associated with nocturnal hypoxia. He underwent surgery to repair the AVF, which successfully improved clinical status and laboratory parameters. 

## CASE DESCRIPTION

 The patient was an 86-year-old, white, male ex-smoker with dyslipidemia with a prior history of neuralgia of the trigeminal nerve, prostatism, and nephrolithiasis. He also had a history of coronary artery disease. He had undergone cardiac catheterization in 1986, via a right brachial access, with atheromatosis and 30% stenosis of the right coronary (RC), circumflex (CX), and anterior descending (AD) arteries. He had had endovascular treatment to repair an abdominal aortic aneurysm in 2010 and to repair an aneurysm of the left internal iliac in 2014. 

 In March 2016, he presented with progressive effort dyspnea. In view of suspected non-angina myocardial ischemia, he underwent cardiac catheterization, which revealed atheromatous lesions involving 50% of the proximal third of the AD and 80% of the mid third, 30% of the proximal third of the CX, and 50% of the proximal third, 90% of the mid third, 50% of the distal third of the RC. Angioplasty was conducted in two stages, with a 2-week interval. Diagnostic catheterization and stent angioplasty of the AD and RC were performed, with a total of three accesses, all via the left radial access, since the diagnostic procedure had revealed occlusion of the right brachial artery, related to a prior catheterization. 

 The symptoms improved significantly after angioplasty. However, 1 year after the intervention, the patient began to exhibit symptoms of dyspnea once more, this time unrelated to effort, and underwent investigation again in March 2017. An electrocardiogram revealed a sinusoidal rhythm with a frequency of 57. A stress echocardiogram with ejection fraction at 55% did not reveal evidence of ischemia or fibrosis. Polysomnography found an apnea-hypopnea index (AHI) of 3.8 (0 = apnea and 30 = hypopnea) and oxyhemoglobin saturation varying from 76 to 91% while asleep. Spirometry found forced vital capacity to be within expected limits, airflow mildly reduced, significant variations on the bronchodilator test, and mild obstructive ventilatory disorder. A chest CT found an expanded thoracic aorta, ectatic in the descending portion, with parietal calcifications, atheromatosis of supra-aortic vessels, and ectasia of the trunk of the pulmonary artery. 

 The patient was treated for bronchospasm, with discrete improvement of symptoms. The investigation into the cause of dyspnea was resumed in December 2017, with pulmonary perfusion scintigraphy, which found no significant abnormalities. An echocardiogram conducted in December 2017 revealed concentric left ventricle remodeling, preserved systolic function rated at 65% (Teicholz) and 69% (Simpson), mild mitral valve failure, aortic ectasia, redundant interatrial septum, with no shunting, discrete pulmonary arterial hypertension, with pulmonary artery systolic pressure (PASP) at 36 mmHg and left atrium enlargement. 

 At that time the patient did not exhibit any type of abnormal vital sign, had normal cardiac and pulmonary findings, and 90% saturation in room air. Blood gas analysis results were as follows: pH 7.446; PCO_2_ 34.6; PO_2_ 60.5; bicarbonate 23.3; and oxygen saturation 92.4. All lower limb pulses were present with no significant findings. Distal brachial pulses were absent in the right upper limb, whereas all pulses in the left upper limb were present, with swelling and thrill at the radial pulse. 

**Figure 1:**
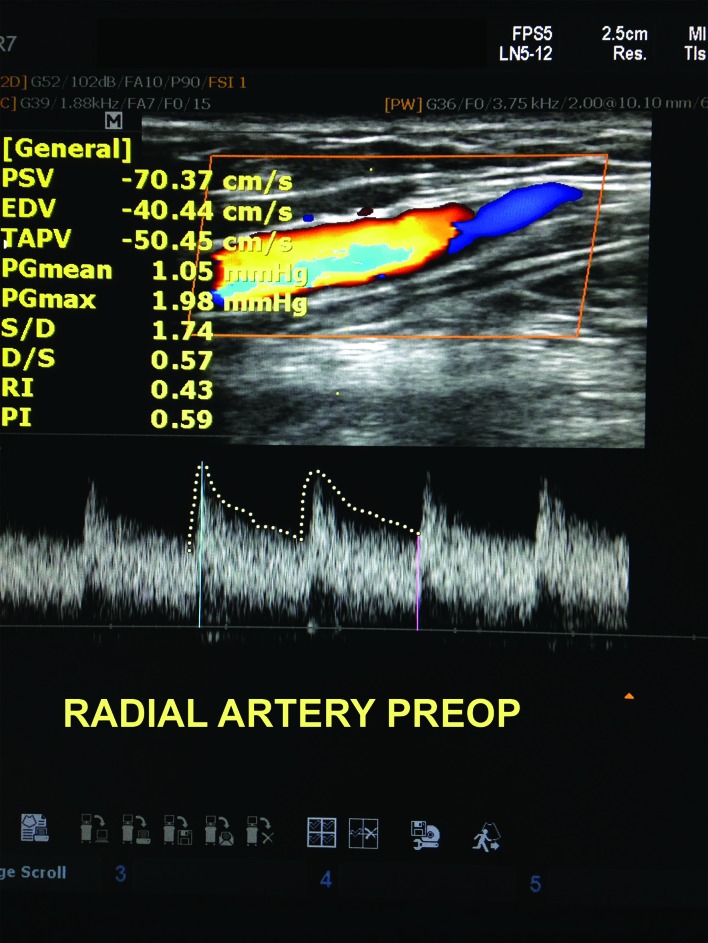
Duplex scan with spectral analysis of the radial artery, proximal to the arteriovenous fistula, before surgery.

**Figure 2:**
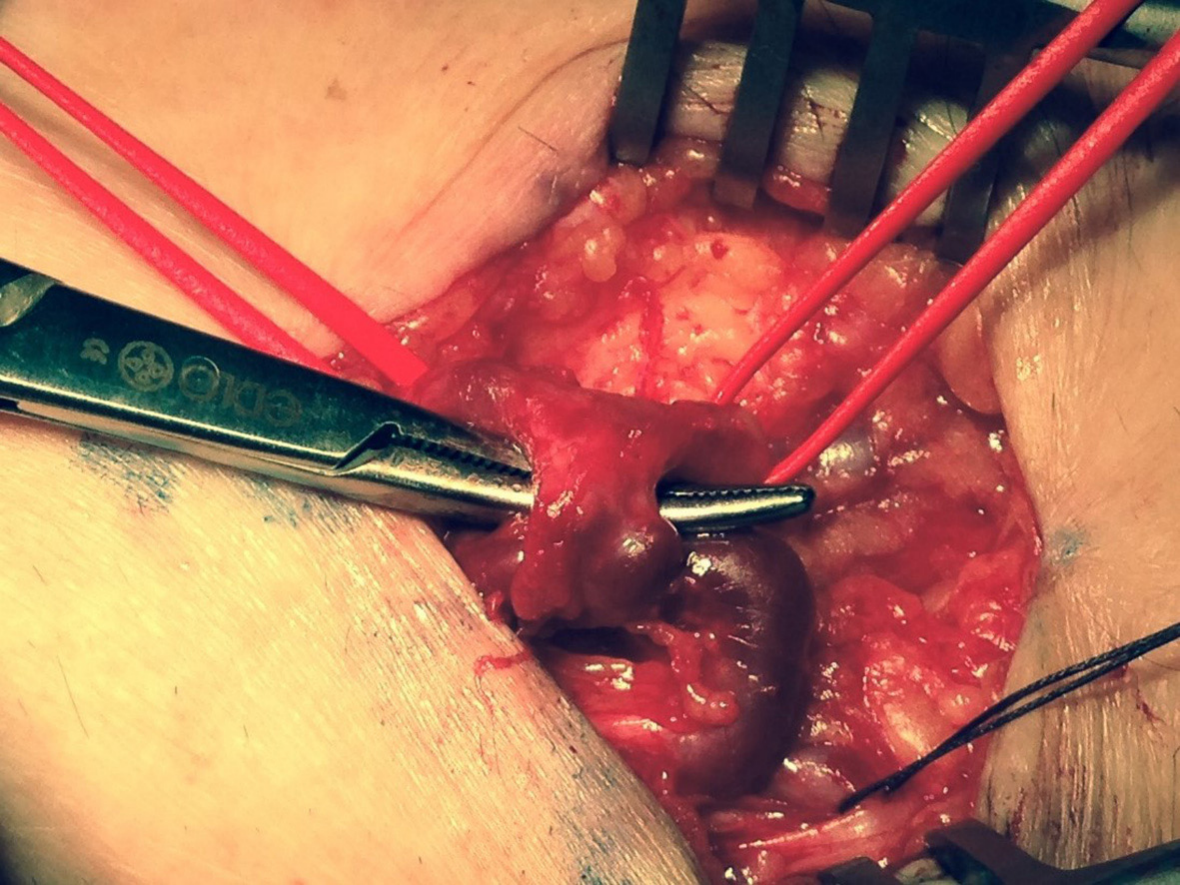
Interoperative image of arteriovenous fistula involving the radial artery and the cephalic vein in the wrist.

 The patient was referred to a vascular surgeon and underwent a duplex scan, which found a fistula between the radial artery and the cephalic vein at the level of the left wrist. Spectral flow analysis of the radial artery revealed a significant increase in diastolic velocity in the segment proximal of the fistula ( Figure 1 ), with reduction of diastolic velocity to normal levels beyond the fistula. In February 2018, the patient underwent surgery under local anesthesia with sedation to repair the fistula ( Figure 2 ). 

**Figure 3:**
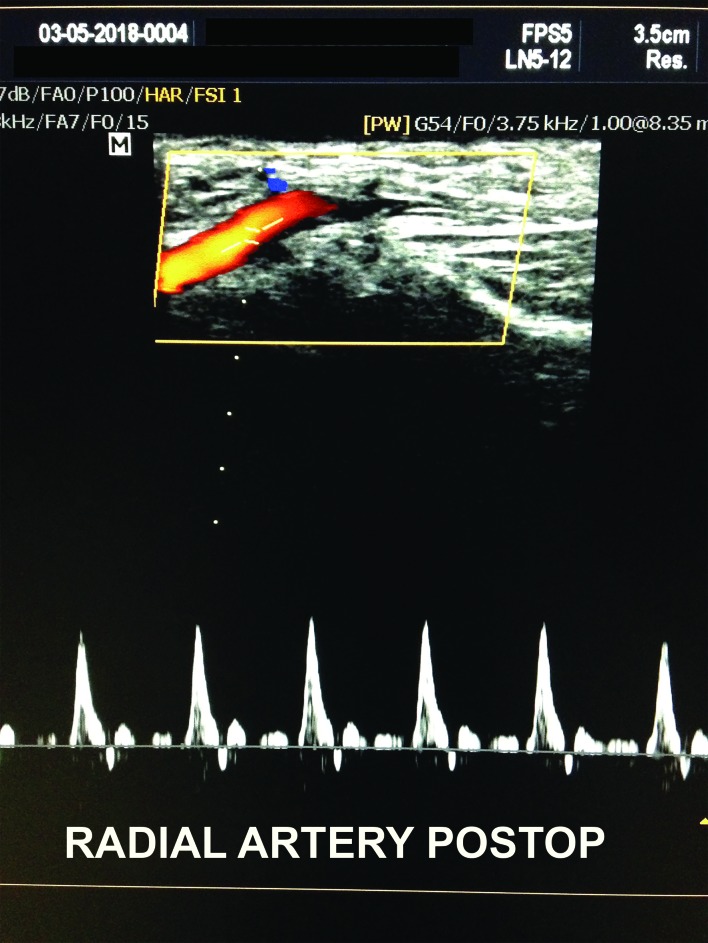
Duplex scan with spectral analysis of the radial artery, proximal of the arteriovenous fistula, after surgery.

 The postoperative period was uneventful, with no complaints. At a 60-day follow-up visit he reported that his dyspnea had improved. A duplex scan showed that diastolic velocity in the segment proximal to the fistula had normalized ( Figure 3 ) and blood gas analysis results were as follows: pH 7.426; PCO_2_ 34; PO_2_ 64.6; bicarbonate 22; and oxygen saturation 93.3. 

## DISCUSSION

 The radial approach for cardiac catheterization offers access to the heart with the same quality as femoral access, while offering certain advantages such as shorter length of hospital stay, earlier mobilization, less bleeding during the procedure and a lower number of complications related to access. 4 The most important disadvantages of radial access tend to be use of greater quantities of contrast and longer duration of the procedure. 2 ^-^ 4

 There is a low rate of conversion from radial to femoral access, at 5.8%, which tends to be associated with factors correlated with arteries of smaller diameter, because of arteriosclerosis or factors inherent to the patient, such as female sex, age over 66 years, disease involving multiple vessels, and patients with small body area. 5 In a recent review of more than 10 thousand cases of radial access for cardiac catheterization, there were vascular or hemorrhagic complications in just 45 (0.44%) patients, just four cases (0.04%) of which were arteriovenous fistulas. 6

 However, the harmful consequences of an AVF in terms of cardiac and pulmonary complications tend to be related to traumatic fistulas that involve large vessels or high-flow AVF, created for dialysis. In these circumstances, the cardiac decompensation provoked by the AVF tends to manifest early, weeks or a few months after the event. 7 ^,^ 8

 In addition to murmur and thrill, if palpable, AVFs with hemodynamic impact tend to manifest with progressive dilation of the veins receiving the arterial blood supply, edema of the extremity involved, and cardiac overload to the point of causing heart failure. However, this characteristic presentation may not occur in up to half of cases, notably in circumstances in which the AVF arises in vessels of medium and small caliber that are farther from the heart. 9

 Heart failure is caused by reduction of peripheral resistance, demanding continuous and, sometimes, increasing effort from the myocardium, attempting to supply the demand caused by the AVF. Iatrogenic fistulas that are not diagnosed promptly tend to exhibit slow progression, with hypertrophy of cardiac chambers and consequent compromise to myocardial functionality, and possibly dysrhythmias such as atrial fibrillation. 10

 In many cases, diagnosis can be made clinically, as in this case. However, work-up tests are useful to quantify the magnitude of the problem and estimate the global impact of the AVF. 

 In the case described here, the symptoms manifest were more the result of the severity of preexisting cardiac and pulmonary diseases than of the magnitude of the AVF, considering the abnormalities found in the supplementary examinations and tests, which were not of great significance. However, the patient’s clinical fragility, which is not rare in octogenarian heart patients, caused a clinical imbalance to the extent that it provoked dyspnea at rest, in the context of the tenuous equilibrium caused by prior diseases. 

## CONCLUSIONS

 Radial access for cardiac catheterization is undoubtedly a safe access route that is capable of offering a lower number of complications, notably with relation to bleeding and vascular injuries. While events such as AVF after radial access are rare, we should be alert to the possibility of their occurrence, particularly when they arise subclinically, since they could be the decisive factor provoking disequilibrium in cardiac or pulmonary diseases that had been under control. 

## References

[1] Kolkailah AA, Alreshq RS, Muhammed AM, Zahran ME, Anas El-Wegoud M, Nabhan AF (2018). Transradial versus transfemoral approach for diagnostic coronary angiography and percutaneous coronary intervention in people with coronary artery disease. Cochrane Database Syst Rev.

[2] Rigattieri S, Sciahbasi A, Ratib K (2016). Comparison between radial approach and femoral approach with vascular closure devices on the occurrence of access. J Invasive Cardiol.

[3] Basu D, Singh PM, Tiwari A, Goudra B (2017). Meta-analysis comparing radial versus femoral approach in patients 75 years and older undergoing percutaneous coronary procedures. Indian Heart J.

[4] Brueck M, Bandorski D, Kramer W, Wieczorek M, Höltgen R, Tillmanns H (2009). A randomized comparison of transradial versus transfemoral approach for coronary angiography and angioplasty. JACC Cardiovasc Interv.

[5] Carvalho MS, Calé R, Gonçalves PA (2015). Predictors of conversion fron radial into femoral Access in cardiac catheterization. Arq Bras Cardiol.

[6] Tatli E, Buturak A, Cakar A (2015). Unusual vascular complications associated with transradial coronary procedures among 10,324 patients: case based experience and treatment options. J Interv Cardiol.

[7] Pilan BF, Oliveira AM, Siqueira DED, Guillaumon AT (2014). Tratamento de fístula arteriovenosa adquirida com repercussões hemodinâmicas graves: desafio terapêutico. J Vasc Bras.

[8] Alkhouli M, Sandhu P, Boobes K, Hatahet K, Raza F, Boobes Y (2015). Cardiac complications of arteriovenous fistulas in patients with end-stage renal disease. Nefrologia..

[9] Wang EA, Lee MH, Wang MC, Lee HY (2004). Iatrogenic left iliac caval fistula: imaging and endovascular treatment. AJR Am J Roentgenol.

[10] Yared K, Baggish AL, Wood MJ (2009). High-output heart failure resulting from a remote traumatic arteriovenous fistula. Can J Cardiol.

