# Supplementary material for: Arteriovenous fistula after radial catheterization with cardiopulmonary repercussions
Source: J Vasc Bras. 2019 Jan 30;18:e20180086. doi: 10.1590/1677-5449.008618 (PMC6542321; doi:10.1590/1677-5449.008618)
Supplement: Translation

# Translation: Fístula arteriovenosa pós-cateterismo radial com repercussões cardiopulmonares

**Authors:** Alexandre Faraco de Oliveira, Alexandre David Ribeiro, Marcio Costa Silveira Ávila

**Affiliations:** 1 Universidade do Planalto Catarinense – UNIPLAC, Lages, SC, Brasil.; 2 Clínica Ana Carolina, Lages, SC, Brasil.

**Keywords:** fístula arteriovenosa, cateterismo cardíaco, intervenção coronária percutânea, dispneia

## Abstract

O presente artigo relata o caso de um paciente coronariopata de 86 anos submetido a cateterismo cardíaco via acesso radial à esquerda. Cerca de 16 meses após o procedimento, manifestou dispneia sem relação com esforço, associada a hipóxia noturna. Apresentava frêmito à palpação do punho esquerdo e foi diagnosticado com fístula arteriovenosa radiocefálica no punho esquerdo. Ao *duplex scan* apresentava alteração de padrão de onda e aumento da velocidade diastólica compatível com fístula arteriovenosa. Foi submetido a correção cirúrgica da fístula, apresentando melhora clínica e laboratorial após o procedimento. O acesso radial para cateterismo cardíaco tem sido cada vez mais utilizado, principalmente por causar complicações menos frequentes e menos deletérias em comparação ao acesso femoral. Entretanto, complicações como fístula arteriovenosa ocorrem e podem ser especialmente prejudiciais em pacientes octogenários.

## INTRODUÇÃO

 O acesso radial com vistas à realização de cateterismo cardíaco, seja diagnóstico ou terapêutico, tem sido cada vez mais utilizado, e hoje é considerado a abordagem preferencial para a maioria das situações 1 . A escolha pela abordagem radial está relacionada principalmente a menor incidência de complicações após o procedimento e a menor sangramento durante o procedimento 2 . 

 Ainda que as complicações relacionadas ao acesso radial sejam de fato menos frequentes, elas não apenas estão presentes como podem ser especialmente danosas para os pacientes mais idosos. Assim, podem determinar uma piora no controle de patologias cardíacas e pulmonares, comumente pré-existentes nesse grupo de pacientes 3 . No presente artigo, descrevemos o caso de um paciente que apresentava coronariopatia, foi submetido a cateterismo cardíaco e angioplastia de coronárias e evoluiu com a formação de uma fístula arteriovenosa (FAV) no punho. Após cerca de 1 ano, manifestou dispneia sem relação com esforço, associada a hipóxia noturna. Foi submetido a cirurgia de correção da FAV, que determinou melhora no quadro clínico e laboratorial associado. 

## DESCRIÇÃO DO CASO

 Um paciente masculino de 86 anos, branco, dislipidêmico e ex-tabagista, apresentava história prévia de neuralgia do trigêmeo, prostatismo e nefrolitíase. Tinha antecedentes de doença arterial coronária. Foi submetido a cateterismo cardíaco em 1986, por acesso braquial direito, com ateromatose e estenose de 30% em coronária direita (CD), circunflexa (CX) e descendente anterior (DA). Foi submetido a tratamento endovascular para correção de aneurisma de aorta abdominal em 2010 e para correção de aneurisma de ilíaca interna esquerda em 2014. 

 Em março de 2016, manifestou dispneia progressiva de esforço. Por suspeita de equivalente anginoso, realizou cateterismo cardíaco, que evidenciou lesão ateromatosa em DA de 50% em terço proximal e 80% em terço médio, CX de 30% em terço proximal, e CD de 50% em terço proximal, 90% em terço médio e 50% em terço distal. Foi realizada angioplastia em dois tempos, com intervalo de duas semanas. Foram realizados cateterismo diagnóstico e angioplastia com stent para DA e para CD, totalizando três acessos, todos por via radial esquerda, uma vez que no procedimento diagnóstico constatou-se oclusão da artéria braquial direita, relacionada ao cateterismo prévio. 

 Houve melhora significativa dos sintomas após a angioplastia. Entretanto, um ano após a intervenção, o paciente voltou a apresentar sintomas de dispneia sem relação com esforço, sendo submetido a nova investigação em março de 2017. Um eletrocardiograma revelou ritmo sinusal com frequência de 57. Um ecocardiograma de estresse com fração de ejeção em 55% não revelou evidências de isquemia ou fibrose. Uma polissonografia mostrou índice de apneia e hipopneia (IAH) de 3,8 (0 apneia e 30 hipopneias) e saturação de oxiemoglobina variando de 76 a 91% durante o sono. Uma espirometria demonstrou capacidade vital forçada dentro dos limites previstos, fluxo aéreo com redução leve, variação significativa ao broncodilatador no teste e distúrbio ventilatório obstrutivo leve. Uma tomografia de tórax revelou aorta torácica alargada, ectásica na porção descendente, com calcificações parietais, ateromatose em vasos supra-aórticos e ectasia do tronco da artéria pulmonar. 

 O paciente foi tratado para broncoespasmo, com melhora discreta dos sintomas. Retomou-se a investigação da causa da dispneia em dezembro de 2017, quando foi realizada cintilografia de perfusão pulmonar, que não apresentou alterações significativas. Um ecocardiograma de dezembro de 2017 revelou ventrículo esquerdo com remodelamento concêntrico, função sistólica preservada em 65% (Teicholz) e 69% (Simpson), insuficiência valvar mitral discreta, ectasia da aorta, septo interatrial redundante, sem shunt, hipertensão arterial pulmonar discreta com pressão sistólica da artéria pulmonar (PSAP) em 36 mmHg e aumento do átrio esquerdo. 

 Apresentou-se naquela data sem qualquer alteração de sinais vitais, com exame cardíaco e pulmonar sem qualquer alteração, saturando 90% em ar ambiente. A gasometria revelou os seguintes valores: pH de 7,446; PCO_2_ de 34,6; PO_2_ de 60,5; bicarbonato de 23,3; e saturação de oxigênio de 92,4. Todos os pulsos estavam presentes nos membros inferiores, sem alterações significativas. No membro superior direito os pulsos braquiodistais estavam ausentes, enquanto no membro superior esquerdo todos os pulsos estavam presentes, com abaulamento e frêmito no pulso radial. 

 Foi encaminhado ao cirurgião vascular e submetido a *duplex scan* , que evidenciou fístula entre a artéria radial e a veia cefálica no nível do punho esquerdo. A análise espectral do fluxo na artéria radial revelou aumento significativo da velocidade diastólica no segmento proximal à fístula ( Figura 1 ), com redução da velocidade diastólica a níveis normais após a fístula. O paciente foi submetido a cirurgia sob anestesia local associada a sedação, em fevereiro de 2018, para correção da fístula ( Figura 2 ). 

**Figura 1:** *Duplex scan* apresentando análise espectral da artéria radial, proximal à fístula arteriovenosa, antes da cirurgia.

**Figura 2:** Transoperatório de fístula arteriovenosa da artéria radial e da veia cefálica no punho.

 Evolui no pós-operatório sem qualquer queixa. No controle de 60 dias após a correção da fístula, referiu melhora da dispneia. Apresentou ao *duplex scan* normalização da velocidade diastólica no segmento proximal à fistula ( Figura 3 ) e gasometria com os seguintes valores: pH de 7,426; PCO_2_ de 34; PO_2_ de 64,6; bicarbonato de 22; e saturação de oxigênio de 93,3. 

**Figura 3:** *Duplex scan* apresentando análise espectral da artéria radial, proximal à fístula arteriovenosa, após a cirurgia.

## DISCUSSÃO

 A abordagem radial para o cateterismo cardíaco permite o acesso ao coração com a mesma qualidade do acesso femoral e apresenta algumas vantagens como tempo de internação menor, deambulação mais precoce, menos sangramento durante o procedimento e menor número de complicações relacionadas ao acesso 4 . As principais desvantagens do acesso radial costumam ser o uso de maior quantidade de contraste e o maior tempo de duração do procedimento 2 ^-^ 4 . 

 A taxa de conversão do acesso radial em femoral também é baixa, de 5,8%, e costuma estar associada a fatores correlacionados a artérias de menor diâmetro pela arteriosclerose ou por questões constitucionais, como sexo feminino, idade maior de 66 anos, doença em múltiplos vasos e pacientes com pequena área corporal 5 . Em uma revisão recente de mais de 10 mil casos de abordagem radial para cateterismo cardíaco, as complicações vasculares ou hemorrágicas aconteceram em apenas 45 (0,44%) pacientes, sendo que apenas quatro casos (0,04%) eram de fístula arteriovenosa 6 . 

 Por outro lado, as consequências deletérias de uma FAV no que se refere a efeitos cardíacos e pulmonares costumam estar associadas às fístulas traumáticas que envolvem grandes vasos ou à FAV de alto fluxo, criada para tratamento dialítico. Nessas circunstâncias, o descontrole cardíaco provocado pela FAV costuma se manifestar precocemente, algumas semanas ou poucos meses após o evento 7 ^,^ 8 . 

 As FAVs com impacto hemodinâmico comumente manifestam – além de sopro e, se palpáveis, frêmito – dilatação progressiva das veias que recebem o aporte de sangue arterial, edema da extremidade envolvida e sobrecarga cardíaca a ponto de causar falência cardíaca. Entretanto, essa apresentação característica pode não ocorrer em até metade dos casos, notadamente nas circunstâncias em que a FAV ocorre em vasos de médio e pequeno calibre, mais distantes do coração 9 . 

 A falência cardíaca se dá em virtude da redução da resistência periférica, exigindo do miocárdio um esforço continuado e, por vezes, crescente, na tentativa de suprir a demanda exigida pela FAV. Fístulas iatrogênicas que não são diagnosticadas precocemente tendem a ter uma evolução arrastada, acarretando hipertrofia de câmaras cardíacas, com consequente prejuízo da funcionalidade do miocárdio, e eventualmente alterações de ritmo como fibrilação atrial 10 . 

 O diagnóstico pode ser realizado clinicamente em muitos casos, como no aqui relatado. Entretanto, os exames complementares são úteis para quantificar a magnitude do problema e estimar o impacto global da FAV. 

 No caso relatado, a sintomatologia apresentada se deve mais à presença e à gravidade das doenças cardíaca e pulmonar prévias do que à magnitude da FAV, tendo em vista as alterações pouco significativas presentes nos exames complementares. Entretanto, a fragilidade clínica do paciente, não rara em cardiopatas octogenários, determinou a ocorrência de um desequilíbrio clínico a ponto de provocar dispneia de repouso, em um contexto de equilíbrio tênue das doenças prévias. 

## CONCLUSÃO

 O acesso radial para cateterismo cardíaco é certamente um acesso seguro e capaz de oferecer menor número de complicações, notadamente no que se refere a sangramento e lesões vasculares. Ainda que eventos como a FAV após abordagem radial sejam raros, devemos estar atentos para a possibilidade de ocorrência, principalmente quando essas situações ocorrem de forma subclínica, podendo ser um fator determinante para o desequilíbrio de doenças cardíacas ou pulmonares anteriormente sob controle.
